# *In vitro* antibacterial activities of *p*-toluenesulfonyl-hydrazinothiazoles and hydrazinoselenazoles against multi-drug resistant Gram-negative phenotypes

**DOI:** 10.1186/s40360-016-0046-0

**Published:** 2016-01-19

**Authors:** Armelle T. Mbaveng, Adriana Grozav Ignat, Bathélémy Ngameni, Valentin Zaharia, Bonaventure T. Ngadjui, Victor Kuete

**Affiliations:** Department of Biochemistry, Faculty of Science, University of Dschang, Dschang, Cameroon; Department of Organic Chemistry, Faculty of Pharmacy, “Iuliu Hatieganu” University of Medicine and Pharmacy, Cluj-Napoca, Romania; Department of Pharmacognosy and Pharmaceutical Chemistry, Faculty of Medicine and Biomedical Sciences University of Yaoundé I, Yaoundé, Cameroon; Department of Organic Chemistry, Faculty of Science, University of Yaoundé I, Yaoundé, Cameroon

**Keywords:** Antibacterial, Efflux pumps, Hydrazinoselenazoles, *p*-toluenesulfonyl-hydrazinothiazoles, Multidrug resistance

## Abstract

**Background:**

Bacterial multidrug resistance (MDR) constitutes a major hurdle in the treatment of infectious diseases worldwide. The present study was designed to evaluate the antibacterial activities of synthetic *p*-toluenesulfonyl-hydrazinothiazoles against multidrug resistant Gram-negative bacteria.

**Methods:**

The broth microdilution method was used to determine the minimal inhibitory concentrations (MIC).

**Results:**

The results demonstrated that the best activities were obtained with hydrazinoselenazoles. *p*-Chloro-benzyliden-selenosemicarbazide, 4-methyl-2-[(4-chloro-benzyliden)-hydrazinyl]-1,3-selenazole, *p*-chloro-benzoyl-selenosemicarbazide and 4-chloromethyl-2-[(4-chlorobenziliden)-*N*-acetyl-hydrazinyl]-1,3-selenazole were more active than the choramphenicol on *Klebsiella pneumoniae* KP63. Tested alone, the lowest MIC value of 16 mg/L was obtained with *p*-methoxy-benzyliden-selenosemicarbazide against *Enterobacter aerogenes* ATCC13048, *K. pneumoniae* ATCC112296 and KP55. Tested in the presence of an efflux pump inhibitor, phenylalanine arginine *β*-naphthylamide (PAβN), the activity of *p*-chloro-benzyliden-selenosemicarbazide, 4-methyl-2-[(4-chloro-benzyliden)-hydrazinyl]-1,3-selenazole, *p*-chloro-benzoyl-selenosemicarbazide and *p*-methoxy-benzyliden-selenosemicarbazide significantly increased with MIC values below 10 mg/L obtained respectively on 43.8 %, 31.3 %, 62.5 % and 100 % of the 16 tested bacterial strains. The lowest MIC value of 0.5 mg/L in the presence of PAβN was recorded with *p*-chloro-benzoyl-selenosemicarbazide against *Escherichia coli* ATCC8739 and KP55 as well as *p*-methoxy-benzyliden-selenosemicarbazide against *E. aerogenes* KP55. *p*-Chloro-benzyliden-selenosemicarbazide and *p*-chloro-benzoyl-selenosemicarbazide contained the same pharmacophore as *p*-methoxy-benzyliden-selenosemicarbazide.

**Conclusion:**

This study indicates that *p*-chloro-benzyliden-selenosemicarbazide, *p*-chloro-benzoyl-selenosemicarbazide and *p*-methoxy-benzyliden-selenosemicarbazide could be explored more to develop novel antimicrobial drugs to fight MDR bacterial infections.

## Background

Bacterial multidrug resistance (MDR) constitutes a major hurdle in the treatment of infectious diseases worldwide. Bacterial efflux systems, especially AcrAB-TolC pumps in *Enterobacteriaceae* or MexAB-OprM in *Pseudomonas aeruginosa*, are involved in multidrug resistance of pathogenic Gram-negative bacteria [1–5]. Clinically, the continuous emergence of Gram-negative MDR bacteria drastically reduces the efficacy of the antibiotic arsenal leading globally to an increase of the frequency of therapeutic failure [6]. Consequently, new antibacterials are needed to fight these bacterial pathogens, but progress in developing them has been slow [7]. *p*-Toluenesulfonyl-hydrazinothiazoles and hydrazinoselenazoles are synthetic compounds with established anticancer, analgesic and anti-inflammatory activities [8–11]. The aim of the present work was to determine the antibacterial activities of a panel of *p*-toluenesulfonyl-hydrazinothiazoles and hydrazinoselenazoles against different bacterial strains expressing MDR phenotype. Furthermore, we highlighted possible pharmacophoric cores amongst the active compounds.

## Methods

### Chemicals for antimicrobial assay

**Fig. 1:**
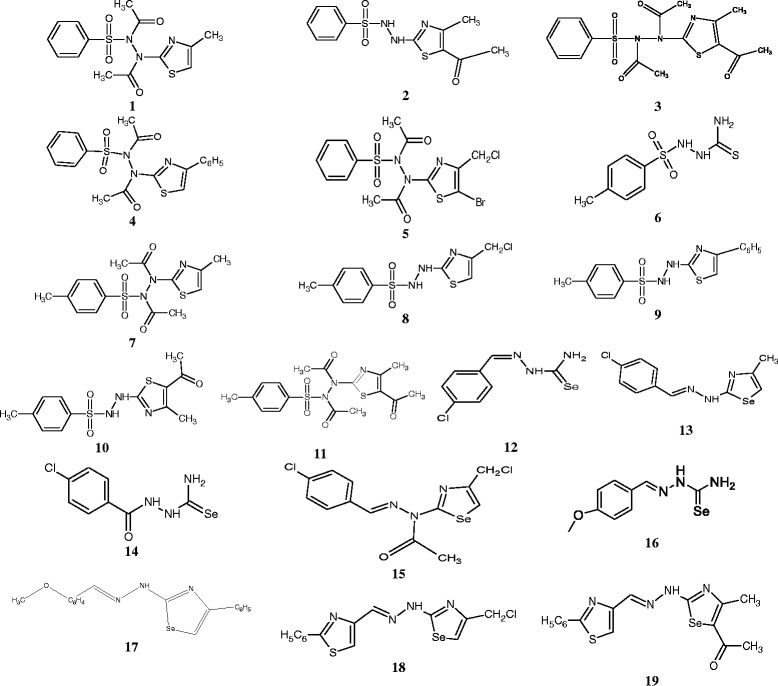
Chemical structures of the tested compounds

Chloramphenicol ≥ 98 % (Sigma-Aldrich, St. Quentin Fallavier, France) was used as reference antibiotics (RA) against the tested bacteria. *p*-iodonitrotetrazolium chloride (INT) and phenylalanine arginine β-naphthylamide (PAβN) were used as microbial growth indicator and efflux pumps inhibitor (EPI) respectively [12, 13]. The tested synthetic compounds included eleven *p*-toluenesulfonyl-hydrazinothiazoles: 4-methyl-2-[2-(benzenesulfonyl)-*N,N*-diacetyl-hydrazino]-thiazole, C_15_H_18_N_3_O_4_S_2_ (**1**; *m/z* 368.07); 5-acetyl-4-methyl-2-[2-(benzenesulfonyl)-hydrazino]-thiazole, C_13_H_18_N_3_O_4_S_2_ (**2**; *m/z* 344.07); 5-acetyl-4-methyl-2-[2-(benzenesulfonyl)-*N,N*-diacetyl-hydrazino]-thiazole, C_17_H_20_N_3_O_5_S_2_ (**3**; *m/z* 410.08); 4-Phenyl-2-[2-(benzenesulfonyl)-*N,N*-diacetyl-hydrazino]-thiazole, C_22_H_24_N_3_O_2_S_2_, (**4**; *m/z* 426.13); 5-bromo-4-Chloromethyl-2-[2-(benzenesulfonyl)-*N,N*-diacetyl-hydrazino]-thiazole, C_17_H_20_BrClN_3_O_2_S_2_ (**5**; *m/z* 475.98); *p*-toluenesulfonylthiosemicarbazide, C_10_H_18_N_3_O_2_S_2_ (**6**; *m/z* 276.08); 4-methyl-2-[2-(*p*-toluenesulfonyl)-*N,N*-diacetyl-hydrazino]-thiazole, C_16_H_20_N_3_O_4_S_2_ (**7**; *m/z* 382.08); 4-chloromethyl-2-[2-(*p*-toluenesulfonyl)-hydrazino]-thiazole, C_12_H_15_ClN_3_O_2_S_2_ (**8**; *m/z* 332.02); 4-phenyl-2-[2-(*p*-toluenesulfonyl)-hydrazino]-thiazole, C_17_H_18_N_3_O_2_S_2_ (**9**; *m/z* 360.08); 5-acetyl-4-methyl-2-[2-(*p*-toluenesulfonyl)-hydrazino]-thiazole, C_14_H_18_N_3_O_3_S_2_ (**10**; *m/z* 340.07); 5-acetyl-4-methyl-2-[2-(*p*-toluenesulfonyl)-*N,N*-diacetylhydrazino]-thiazole, C_18_H_22_N_3_O_5_S_2_ (**11**; *m/z* 424.10) and hydrazinoselenazoles: *p*-chloro-benzyliden-selenosemicarbazide, C_8_H_8_ClN_3_Se (**12**; *m/z* 260.95); 4-methyl-2-[(4-chloro-benzyliden)-hydrazinyl]-1,3-selenazole, C_11_H_10_ClN_3_Se (**13**; *m/z* 298.97,); *p*-chloro-benzoyl-selenosemicarbazide C_8_H_8_ClN_3_OSe (**14**; *m/z* 276.95); 4-chloromethyl-2-[(4-chlorobenziliden)-*N*-acetyl-hydrazinyl]-1,3-selenazole, C_13_H_11_Cl_2_N_3_OSe (**15**; *m/z* 374.94); *p*-methoxy-benzyliden-selenosemicarbazide, C_9_H_11_N_3_OSe (**16**; *m/z* 257.00); 4-phenyl-[2-(4-metoxybenzyliden)-hydrazinyl]-1,3-selenazole, C_17_H_15_N_3_OSe (**17**; *m/z* 357.03); 4-chloromethyl-[2-(2-phenyl-1,3-thiazolo-4-methylidene)-hydrazinyl]-1,3-selenazole C_16_H_19_ClN_4_SSe (**18**; *m/z* 414.01); and 4-methyl-5-acetyl-[2-(2-phenyl-1,3-thiazolo-4-methylidene)-hydrazinyl]-1,3-selenazole, C_18_H_22_N_4_OSSe (**19**; *m/z* 422.06). The synthesis of *p*-toluenesulfonyl-hydrazinothiazoles (**1**–**11**) [9] and hydrazinoselenazole (**12**–**19**) [11] were previously reported. Their chemical structures are summarized in Fig. 1.

### Microbial strains and culture media

The studied microorganisms included sensitive and resistant strains of *Pseudomonas aeruginosa, Klebsiella pneumoniae, Enterobacter aerogenes, Escherichia coli* obtained from the American Type Culture Collection (ATCC) as well as clinical strains. Their bacterial feature were previously reported [5, 14–16]. Nutrient agar were used for the activation of the tested Gram-negative bacteria [17].

### INT colorimetric assay for MIC and MBC determinations

The Minimum Inhibitory Concentration (MIC) determinations on the tested bacteria were conducted using rapid *p*-iodonitrotetrazolium chloride (INT) colorimetric assay according to described methods [12] with some modifications [18, 19]. The tested samples and RA were first of all dissolved in DMSO/Mueller Hinton Broth (MHB). The final concentration of DMSO was lower than 2.5 % and does not affect the microbial growth [20, 21]. The solution obtained was then added to Mueller Hinton Broth, and serially diluted two fold (in a 96- wells microplate). One hundred microlitre (100 μL) of inoculum 1.5 x 10^6^ CFU/mL prepared in appropriate broth was then added [18, 19]. The plates were covered with a sterile plate sealer, then agitated to mix the contents of the wells using a plate shaker and incubated at 37 °C for 18 h. The assay was repeated thrice. Wells containing adequate broth, 100 μL of inoculum and DMSO to a final concentration of 2.5 % served as negative control. The MIC of samples was detected after 18 h incubation at 37 °C, following addition (40 μL) of 0.2 mg/mL of INT and incubation at 37 °C for 30 min. Viable bacteria reduced the yellow dye to a pink. MIC was defined as the sample concentration that prevented the color change of the medium and exhibited complete inhibition of microbial growth [12]. To evaluate the role of efflux pumps in the susceptibility of Gram-negative bacteria, compounds were tested in the presence of PAßN (at 30 μg/mL) and MIC was determined as above.

## Results

### Studied compounds

Compounds tested in the present study included *p*-toluenesulfonyl-hydrazinothiazoles (**1**–**11**) [9] and hydrazinoselenazoles (**12**–**19**) [11] previously synthesized. They were tested for their antimicrobial activities on a panel of 16 bacterial strains and the results are summarized in Table 1.

**Table 1:** Minimal inhibitory concentration (MIC) of the studied samples on the tested microbial species

Samples					*E. coli* strains and MIC* (mg/L) without PAβN (and with PAβN in parenthesis)												% of PAβN Modulating effect
	*E. coli* strains						*E. aerogenes* strains					*K. pneumoniae*			*P. aeruginosa*		
	ATCC87 39	AG100	AG100A	AG100A tet	AG102	ATCC 13048	EACM64	EA289	EA294	EA298	EA27	ATCC 11296	KP55	KP63	PA01	PA124	
***p*** **-Toluenesulfonylhydrazinothiazoles**																	
**1**	i	i	i	i	i	i	i	i	i (64)	i (256)	i	i	i	i	i	i	2/16 (12.5%)
**2**	i	i	i	i	i	i	i	i	i (64)	i	i	i	i	i	i	i	1/16 (6.3%)
**3**	i (256)	i	i	i	i	i	i	i	i (64)	i (256)	i	i 256 (256)	i	i	i	i	3/16 (18.8%)
**4**	i	i	i	i	i	i	i	i	i (64)	i (32)	i	i	i	i	i	i	2/16 (12.5%)
**5**	i	i	i	i	i	i	i	i	i (64)	i	i	i	i	i	i	i	1/16 (6.3%)
**6**	i	i	i	i	i	i	i	i	i (64)	i	i	i 256 (256)	i	i	i	i	1/16 ( 6.3%)
**7**	i	i	i	i	i	i	i	i	i (64)	i (256)	i	i (256)	i (256)	i	i	i	4/16 (25.0 %)
**8**	256 (256)	256 (256)	256 (256)	128 (128)	256 (256)	256 (256)	256 (256)	256 (256)	256 (256)	i (256)	256 (256)	256 (256)	256 (256)	256 (256)	i	256 (256)	1/16 (6.25 %)
**9**	i	i	i	i	i	i	i	i	i (64)	i	i	i	i	i	i	i	1/16 (6.25%)
**10**	i	i	i	i	i	i	i	i	i	i	i	i	i	i	i	i	0/16 ( 0%)
**11**	i (256)	i	i	i	i	i	i	i	i	i	i	i (256)	i	i (256)	i	i	3/16 ( 18.75%)
**Hydrazinoselenazoles**																	
**12**	256 (**4**)	256 (64)	i (**4**)	i (16)	i (16)	i (256)	i (16)	256 (128)	i (**8**)	i (**8**)	i (128)	i (**8**)	128 (**4**)	128 (**4**)	i	i (256)	**15/16 (93.8%)**
**13**	256 (16)	128 (**8**)	128 (**4**)	128 (64)	i	i	i	i	i (**8**)	i (**8**)	i	128 (64)	128 (16)	32 (**8**)	i	i	9/16 (56.3%)
**14**	256 (**0.5**)	i (256)	i (**1**)	i (**2**)	i (**8**)	i (32)	i (**8**)	i (32)	i (**2**)	i (**2**)	i (64)	256 (**2**)	64 (**0.5**)	128 (**1**)	i (256)	i (256)	**16/16 ( 100%)**
**15**	i	i	i	i	i	i	i	i	i	i	i	256 (64)	32 (32)	128 (32)	i	i (256)	3/16 (18.75 %)
**16**	64 (**2**)	256 (**1**)	256 (**2**)	64 (**8**)	64 (**8**)	16 (**8**)	128 (**8**)	256 (**8**)	i (**2**)	i (**2**)	256 (**8**)	16 (**2**)	16 (**0.5**)	32(**1**)	128 (**8**)	128 (**8**)	**16/16 (100 %)**
**17**	i	i	i	i (256)	**i**	i	i	i	i (32)	i (32)	**i**	i 256 (16)	i (32)	i	i	i (128)	6/16 (37.5%)
**18**	i	i	i (128)	i	i	i	i	i	i (16)	i (32)	i	i (32)	256 (32)	i	i	i	5/16 (25.8 %)
**19**	i 256 (128)	i	256 (128)	256 (128)	256 (256)	i	i	i (128)	256 (32)	256 (32)	i (128)	256 (16)	256 (32)	i (256)	i	i (256)	11/16 ( 68.8%)
**Chloramphenicol**	**4** (**1**)	**4** (**0.5**)	**0.5** (**0.25**)	**32** (**4**)	**32** (**2**)	**4** (**1**)	256 (**8**)	i (128)	64 (16)	64 (16)	i (128)	**4** (**1**)	32 (**4**)	i (128)	128 (**8**)	256 (**8**)	**16/16 (100 %)**

*The MIC of PAßN was 64 mg/L on AG100A and >256 mg/L for other *E. coli* , *K. pneumoniae*, *P. aeruginosa* and *E*. aerogenes strains

i: sample not active up to 256 mg/L; (): sample tested in presence of PAßN at 20 mg/L final concentration; (-): not tested; In bold: significant activity

### Activity of p-toluenesulfonyl-hydrazinothiazoles

When they were tested alone at up to 256 mg/L, all *p*-toluenesulfonyl-hydrazinothiazoles generally displayed low or no antibacterial activity. However, compound **8** displayed a best activity with detectable MIC values against 14/16 (87.5 %) bacterial strains (Table 1). This compound was also more active than chloramphenicol against *E. aerogenes* EA289, EA27 and *K. pneumoniae* KP63. No detectable MIC value was recorded with compounds **1, 2, 4, 5, 7, 9, 10** and **11** (Table 1).

### Activity of hydrazinoselenazoles

The best activities were obtained with *p*-toluenesulfonyl-hydrazinothiazoles; When they were tested at up to 256 mg/L, hydrazinoselenazole displayed selective antibacterial activities. Compound **16** displayed MIC values on 14/16 (87.5 %) tested bacteria whilst compounds **12, 13, 14, 15, 19** and were active respectively on 5/16 (31.3 %), 7/16 (43.8 %), 4/16 (25.0 %), 3/16 (18.8 %) and 7/16 (43.8 %) and tested bacteria (Table 1). Compounds **12, 13, 14** and **15** were more active (lower MIC value) than the reference drug (choramphenicol) on *K. pneumoniae* KP63. Compounds **12** was also more active than the reference drug on *E. aerogenes* EA289 meanwhile this was also the case with **16** against *E. aerogenes* CM64, EA27, EA289, KP55 and KP63. Tested alone, the lowest MIC value of 16 mg/L was obtained with compound **16** against *E. aerogenes* ATCC13048, *K. pneumoniae* ATCC11296 and KP55 (Table 1).

### Role of efflux pumps in the susceptibility of tested bacteria: identification of a PAβN-sensitive efflux in addition to AcrAB-TolC pump

To evaluate the role of efflux on the susceptibility of bacteria to the tested compounds, they were combined with a well-known EPI, PAβN. The EPI was tested at 30 μg/mL, a concentration lower that it MIC value in the studied bacteria. The results are summarized in brackets in Table 1 (MIC of EPI is provided as footnote). Apart from *p*-toluenesulfonyl-hydrazinothiazole **10**, PAβN improved the activity of all tested compounds on at least one tested bacteria. The most important improvements of the activity of compounds in the presence of PAβN were recorded with **12, 14, 16** and **19** on respectively 15/16 (93.8 %), 16/16 (100 %), 16/16 (100 %) and 11/16 (68.8 %) tested bacteria. With PAβN, the activity of hydrazinoselenazoles **12, 13, 14** and **16** significantly increased with MIC values below 10 mg/L obtained respectively on 7/16 (43.8 %), 5/16 (31.3 %), 10/16 (62.5 %) and 16/16 (100 %) tested bacterial strains. The lowest MIC value of 0.5 mg/L was recorded with compounds **14** against *E. coli* ATCC8739 and KP55 as well as **16** against *E. aerogenes* KP55 (Table 1). When they were combined with EPI, hydrazinoselenazoles **12, 13, 14** and **16** were more active than choramphenicol on at least two of the tested bacterial species.

### Structure activity relationship and identification of pharmacophoric moiety

**Fig. 2:**
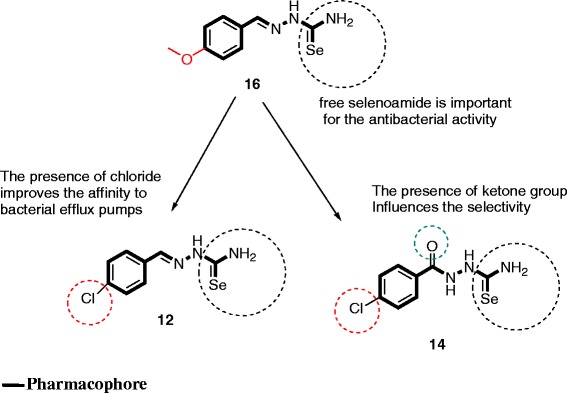
Representation of pharmacophoric groups detected in the tested compounds

When regarding the structure-activity relationship, it appears that amongst the tested compounds, *p*-toluenesulfonyl-hydrazinothiazoles were less active than hydrazinoselenazoles. Within *p*-toluenesulfonyl-hydrazinothiazoles, it was also found that compound **8** scaffold is the most active and that any modification of its structure resulted to a decrease of antibacterial activity. Within hydrazinoselenazoles, it was also found that compound **16** [active on 14/16 (87.5 %) and 16/16 (100 %) tested bacteria in the absence or presence of EPI respectively; MIC values below 10 mg/L on all tested bacteria in the presence of EPI and lowest MIC value of 0.5 mg/L] can be considered as the phamacophoric moiety. Within this moiety, the presence of a free selenoamide is primordial for the antibacterial activity, as compounds **12** and **14** (bearing a free selenoamide**)** were also very active, especially when they were combined with EPI (Fig. 2). The presence of chloride substituent seems to increase the affinity of hydrazinoselenazole **12** and **14** to bacteria efflux pumps. In fact in the absence of EPI, the activity of compounds **12** and **14** (with chloride substituent) were much lower than that of **16** (with -OCH_3_ substituent**)**.

## Discussion

Bacterial multidrug resistance represents a major problem in the treatment of infectious diseases. In the present study, we tested a panel of bacterial strains including both reference ATCC strains and MDR phenotypes expressing active efflux pumps [4, 5, 22]. In fact, tripartite drug efflux pumps, mainly those clinically reported as AcrAB–TolC in Enterobacteriaceae or as MexAB–OprM in *P. aeruginosa* tested in the present study, play a key role in multidrug resistance of pathogenic Gram-negative bacteria [23, 24]. *p*-Toluenesulfonyl-hydrazinothiazoles and hydrazinoselenazoles are synthetic compounds that previously displayed good anticancer, analgesic and anti-inflammatory activities [8–11]. Herein, we investigated the ability of such compounds to combat MDR Gram-negative bacteria expressing active efflux pumps. As results, it was found that hydrazinoselenazoles were much more active than *p*-toluenesulfonyl-hydrazinothiazoles. However, all the tested chemicals were substrates of bacterial efflux pumps. Interestingly, when efflux pumps were blocked by EPI, significant increase of the activity of three compounds **12, 14** and **16** was observed. More importantly, the obtained activity was better than that of the reference drug chloramphenicol in many cases (Table 1), suggesting that the three compounds can be used in combination with PAβN to fight bacterial infections involving MDR phenotypes. The antimicrobial activity of a compound has been defined as significant when MIC is below 10 mg/L, moderate when 10 mg/L < MIC < 100 mg/L or low when MIC > 100 mg/L [25, 26]. In this study, the MIC values below 10 mg/L was noted with hydrazinoselenazoles **12, 14** and **16** on 43.8 %, 62.5 % and 100 % tested bacterial strains when they were tested in the presence of EPI, highlighting their possible use in combination with PAβN in the control of MDR bacterial infections.

Regarding the involvement of MDR bacteria in treatment failures and the re-emergence of infectious diseases [1, 27, 28], these findings can be considered very promising. In fact, *Pseudomonas aeruginosa* is an important nosocomial pathogen highly resistant to clinically used antibiotics, causing a wide spectrum of infections and leading to substantial morbidity and mortality [29] and was found sensitive to the three compounds. MDR Enterobacteriaceae, including *K. pneumoniae*, *E. aerogenes, P. stuartii* and *E. coli*, have also been classified as antimicrobial-resistant organisms of concern in healthcare facilities [28, 30].

## Conclusions

Regarding the medical importance of the studied microorganisms, the results obtained in this study demonstrated that hydrazinoselenazoles are strong source of inspiration in antibacterial drug discovery. Thus, the present data showed that hydrazinoselenazoles **12, 14** and **16** are promising antibacterial agents to combat MDR phenotypes, but are also substrates of efflux pumps. They should therefore be combined with an EPI to combat MDR phenotypes. We also found that compound **16** skeleton was the pharmacophoric moiety of the tested hydrazinoselenazoles and that the presence of a free selenoamide scaffold is necessary for their antibacterial activity. However, more compounds should be further synthesized to fully investigate the structure-activity relationship for this series of hydrazinoselenazoles. Toxicological studies will also be performed to ensure the safety of the active hydrazinoselenazoles, especially compound **16**.

## Abbreviations

**1**: 4-methyl-2-[2-(benzenesulfonyl)-*N,N*-diacetyl-hydrazino]-thiazole
**2**: 5-acetyl-4-methyl-2-[2-(benzenesulfonyl)-hydrazino]-thiazole
**3**: 5-acetyl-4-methyl-2-[2-(benzenesulfonyl)-*N,N*-diacetyl-hydrazino]-thiazole
**4**: 4-phenyl-2-[2-(benzenesulfonyl)-*N,N*-diacetyl-hydrazino]-thiazole
**5**: 5-bromo-4-Chloromethyl-2-[2-(benzenesulfonyl)-*N,N*-diacetyl-hydrazino]-thiazole
**6**: *p*-toluenesulfonylthiosemicarbazide
**7**: 4-methyl-2-[2-(*p*-toluenesulfonyl)-*N,N*-diacetyl-hydrazino]-thiazole
**8**: 4-chloromethyl-2-[2-(*p*-toluenesulfonyl)-hydrazino]-thiazole
**9**: 4-phenyl-2-[2-(*p*-toluenesulfonyl)-hydrazino]-thiazole
**10**: 5-acetyl-4-methyl-2-[2-(*p*-toluenesulfonyl)-hydrazino]-thiazole
**11**: a-Acetyl-4-methyl-2-[2-(*p*-toluenesulfonyl)-*N,N*-diacetyl-hydrazino]-thiazole
**12**: *p*-chloro-benzyliden-selenosemicarbazide
**13**: 4-methyl-2-[(4-chloro-benzyliden)-hydrazinyl]-1,3-selenazole
**14**: *p*-chloro-benzoyl-selenosemicarbazide
**15**: 4-chloromethyl-2-[(4-chlorobenziliden)-N-acetyl-hydrazinyl]-1,3-selenazole
**16**: *p*-methoxy-benzyliden-selenosemicarbazide
**17**: 4-phenyl-[2-(4-metoxybenzyliden)-hydrazinyl]-1,3-selenazole
**18**: 4-chloromethyl-[2-(2-phenyl-1,3-thiazolo-4-methylidene)-hydrazinyl]-1,3-selenazole
**19**: 4-methyl-5-acetyl-[2-(2-phenyl-1,3-thiazolo-4-methylidene)-hydrazinyl]-1,3-selenazole
ATCC: American Type Culture Collection
CFU: Colony forming unit
DMSO: dimethysulfoxide
EPI: Efflux pumps inhibitor
INT: *p*-iodonitrotetrazolium chloride
MDR: Multidrug resistant
MHB: Mueller Hinton Broth
MIC: Minimal inhibitory concentration
PAβN: Phenylalanine arginine *β*-naphthylamide
RA: Reference antibiotic

## References

[1] Blot S, Depuydt P, Vandewoude K, De Bacquer D (2007). Measuring the impact of multidrug resistance in nosocomial infection. Curr Opin Infect Dis.

[2] Pietras Z, Bavro VN, Furnham N, Pellegrini-Calace M, Milner-White EJ, Luisi BF (2008). Structure and mechanism of drug efflux machinery in Gram negative bacteria. Curr Drug Targets.

[3] Papadopoulos CJ, Carson CF, Chang BJ, Riley TV (2008). Role of the MexAB-OprM efflux pump of Pseudomonas aeruginosa in tolerance to tea tree (Melaleuca alternifolia) oil and its monoterpene components terpinen-4-ol, 1,8-cineole, and alpha-terpineol. Appl Environ Microbiol.

[4] Kuete V, Ngameni B, Tangmouo JG, Bolla JM, Alibert-Franco S, Ngadjui BT (2010). Efflux pumps are involved in the defense of Gram-negative bacteria against the natural products isobavachalcone and diospyrone. Antimicrob Agents Chemother.

[5] Kuete V, Alibert-Franco S, Eyong KO, Ngameni B, Folefoc GN, Nguemeving JR (2011). Antibacterial activity of some natural products against bacteria expressing a multidrug-resistant phenotype. Int J Antimicrob Agents.

[6] Rice LB (2006). Unmet medical needs in antibacterial therapy. Biochem Pharmacol.

[7] Fischbach MA, Walsh CT (2009). Antibiotics for emerging pathogens. Science.

[8] Ignat A, Zaharia V, Mogosan C, Palibroda N, Cristea C, Silaghi-Dumitrescu L (2010). Heterocycles 25. Microwave assisted synthesis of some *p-*toluensulfonylhydrazinothiazoles with analgesic and anti-inflammatory activity. Farmacia.

[9] Zaharia V, Ignat A, Palibroda N, Ngameni B, Kuete V, Fokunang CN (2010). Synthesis of some p-toluenesulfonyl-hydrazinothiazoles and hydrazino-bis-thiazoles and their anticancer activity. Eur J Med Chem.

[10] Ignat Grozav A, Gaina L, Kuete V, Silaghi-Dumitrescu L, Efferth T, Zaharia V (2013). Microwave-assisted synthesis of new selenazole derivatives with antiproliferative activity. Molecules.

[11] Zaharia V, Ignat A, Ngameni B, Kuete V, Moungang M, Fokunang C (2013). Heterocycles 23: Synthesis, characterization and anticancer activity of new hydrazinoselenazole derivatives. Med Chem Res.

[12] Eloff JN (1998). A sensitive and quick microplate method to determine the minimal inhibitory concentration of plant extracts for bacteria. Planta Med.

[13] Mativandlela SPN, Lall N, Meyer JJM (2006). Antibacterial, antifungal and antitubercular activity of (the roots of) *Pelargonium reniforme* (CURT) and *Pelargonium sidoides* (DC) (Geraniaceae) root extracts. S Afr J Bot.

[14] Lacmata ST, Kuete V, Dzoyem JP, Tankeo SB, Teke GN, Kuiate JR (2012). Antibacterial activities of selected Cameroonian plants and their synergistic effects with antibiotics against bacteria expressing MDR phenotypes. Evid Based Complement Alternat Med.

[15] Seukep JA, Fankam AG, Djeussi DE, Voukeng IK, Tankeo SB, Noumdem JA (2013). Antibacterial activities of the methanol extracts of seven Cameroonian dietary plants against bacteria expressing MDR phenotypes. Springerplus.

[16] Touani FK, Seukep AJ, Djeussi DE, Fankam AG, Noumedem JA, Kuete V (2014). Antibiotic-potentiation activities of four Cameroonian dietary plants against multidrug-resistant Gram-negative bacteria expressing efflux pumps. BMC Complement Altern Med.

[17] Kuete V, Kamga J, Sandjo LP, Ngameni B, Poumale HM, Ambassa P (2011). **Antimicrobial activities of the methanol extract, fractions and compounds from*****Ficus polita*****Vahl. (Moraceae)**. BMC Complement Altern Med.

[18] Kuete V, Nana F, Ngameni B, Mbaveng AT, Keumedjio F, Ngadjui BT (2009). Antimicrobial activity of the crude extract, fractions and compounds from stem bark of *Ficus ovata* (Moraceae). J Ethnopharmacol.

[19] Kuete V, Wansi JD, Mbaveng AT, Kana Sop MM, Tadjong AT, Beng VP (2008). Antimicrobial activity of the methanolic extract and compounds from *Teclea afzelii* (Rutaceae). S Afr J Bot.

[20] Kuete V, Ngameni B, Simo CC, Tankeu RK, Ngadjui BT, Meyer JJ (2008). Antimicrobial activity of the crude extracts and compounds from *Ficus chlamydocarpa* and *Ficus cordata* (Moraceae). J Ethnopharmacol.

[21] Kuete V, Wabo GF, Ngameni B, Mbaveng AT, Metuno R, Etoa FX (2007). Antimicrobial activity of the methanolic extract, fractions and compounds from the stem bark of *Irvingia gabonensis* (Ixonanthaceae). J Ethnopharmacol.

[22] Fankam AG, Kuete V, Voukeng IK, Kuiate JR, Pages JM (2011). Antibacterial activities of selected Cameroonian spices and their synergistic effects with antibiotics against multidrug-resistant phenotypes. BMC Complement Altern Med.

[23] Nikaido H (2009). Multidrug resistance in bacteria. Annu Rev Biochem.

[24] Davin-Regli A, Bolla JM, James CE, Lavigne JP, Chevalier J, Garnotel E (2008). Membrane permeability and regulation of drug “influx and efflux” in enterobacterial pathogens. Curr Drug Targets.

[25] Kuete V (2010). Potential of Cameroonian plants and derived products against microbial infections: a review. Planta Med.

[26] Kuete V, Efferth T (2010). Cameroonian medicinal plants: pharmacology and derived natural products. Front Pharmacol.

[27] Falagas ME, Bliziotis IA (2007). Pandrug-resistant Gram-negative bacteria: the dawn of the post-antibiotic era?. Int J Antimicrob Agents.

[28] Nicolle L: Infection control programmes to contain antimicrobial resistance. Geneva: World Health Organization. http://whqlibdoc.who.int/hq/2001/WHO_CDS_CSR_DRS_2001.7.pdf. [accessed October 2014].

[29] Cardoso O, Alves AF, Leitao R (2007). Surveillance of antimicrobial susceptibility of *Pseudomonas aeruginosa* clinical isolates from a central hospital in Portugal. J Antimicrob Chemother.

[30] Tran QT, Mahendran KR, Hajjar E, Ceccarelli M, Davin-Regli A, Winterhalter M (2010). Implication of porins in beta-lactam resistance of *Providencia stuartii*. J Biol Chem.

